# Transcriptomic characterization of mesenchymal and skin tissues in *Cervus elaphusxanthopygus* antler and identification of growth-related candidate genes

**DOI:** 10.7717/peerj.21606

**Published:** 2026-07-27

**Authors:** Xiaodan Bi, Bing Liu, Bing Li, Meirong Zhao, Huimin Tian, Jianxing Chen

**Affiliations:** 1 College of Agriculture of Chifeng University, Chifeng, Inner Mongolia, China; 2 College of Chemistry and Life Sciences of Chifeng University, Chifeng, Inner Mongolia, China

**Keywords:** *Cervus elaphus xanthopygus*, Antlers

## Abstract

**Background:**

Deer antler is the only mammalian organ capable of periodic complete regeneration. Its astonishing growth rate—reaching several centimeters per day—provides a unique model for research in tissue regeneration and developmental biology. This rapid growth relies on the protective and signaling functions of the skin, as well as the vigorous proliferation and differentiation capacity of mesenchymal cells. Although previous studies have identified some key factors involved in antler growth, systematically comparing the transcriptomic profiles of these two core tissues (skin and mesenchyme) to identify candidate genes regulating their synergistic growth is crucial for deciphering the molecular mechanisms underlying this “ultra-fast” growth.

**Methods:**

The Illumina NovaSeq 6000 sequencing platform was used to perform transcriptome sequencing on the antler tissues of the *Cervus elaphus xanthopygus* (Dongbei red deer) with a growth period of approximately 60 days. Bioinformatics methods were used to identify the transcriptional expression of genes in the antler tissues, and the expression levels of some genes were tested by fluorescence quantitative PCR.

**Results:**

A total of 109.82 Gb of transcriptome data was generated from the skin and mesenchymal tissues of antlers, and 40,023 transcripts were obtained. There were 3,530 genes with differential expression between the skin and the mesenchymal tissues of antlers. Genes with upregulated expression levels in the mesenchymal layer were enriched in phosphate-containing compound metabolic processes and multiple biological processes related to bone development such as skeletal system development, cartilage development, regulation of ossification, and enamel mineralization. Among the genes enriched in the cancer pathway and the PI3K-Akt signaling pathway, those that may play an important role in the rapid growth process of deer antlers were screened, including *MDM2*, *RPS6KB1*, *CASP3*, *BMP4*, *PTCH1*, *SOX-6*, *YWHAZ*, and *HSP90B1*.

**Conclusions:**

The discovery of these genes provides a reference for a further in-depth understanding of the mechanism of the rapid growth of antlers.

## Introduction

Deer antler is a unique bony organ that grows on the frontal bones of male cervids. Characterized by its rare capacity for complete regeneration and extremely rapid growth rate in nature, it serves as an ideal model for studying mammalian organ regeneration and rapid tissue growth (Li et al., 2007). During the peak growing season, antlers can elongate at a rate of 1–2 cm per day—an astonishing speed that even surpasses the proliferation rate of most cancer cells. However, this process is strictly genetically regulated and ultimately results in normal bone tissue. Consequently, the antler provides a unique window for elucidating the molecular mechanisms underlying tissue regeneration and ossification.

The growth and development of deer antler is a highly coordinated biological process involving the precise collaboration of multiple tissue layers (Li, Suttie & Clark, 2005). Among these, the mesenchymal layer serves as the core region for rapid growth; it is rich in pluripotent stem cells and actively proliferating progenitor cells (Li et al., 2010; Li, 2012; Qin et al., 2023), which directly drive the longitudinal extension and thickening of the antler bone *via* endochondral ossification. Concurrently, the velvet skin covering the outer layer not only provides nutritional support and mechanical protection but also participates in regulating the proliferation and differentiation of mesenchymal cells by secreting growth factors and cytokines (Turner & Grose, 2010). However, despite the synergistic roles of the mesenchyme and velvet skin in antler development, the gene expression profiles and molecular regulatory networks between these two tissues remain largely unexplored.

In recent years, transcriptome sequencing (RNA-seq) has become a crucial tool for elucidating the molecular mechanisms of complex biological processes, owing to its advantages of high throughput, high sensitivity, and single-base resolution. By comparing transcriptomic differences across various tissues or developmental stages, key regulatory genes and their associated signaling pathways can be effectively identified (Wang et al., 2019). Against this background, this study selected Northeastern red deer at approximately 60 days of antler growth as the research object. At this stage, the antlers are in a phase of vigorous growth, and mesenchymal cell division and proliferation are most active. Using the Illumina NovaSeq 6000 platform, we performed transcriptome sequencing on velvet skin and mesenchymal tissues to systematically identify differentially expressed genes (DEGs). We focused on screening for genes significantly upregulated in the mesenchymal layer and used bioinformatics analysis to explore the biological processes and signaling pathways they may be involved in. The results aim to provide candidate gene resources for deciphering the molecular regulatory mechanisms of rapid antler growth and offer theoretical references for research on mammalian tissue regeneration and bone metabolism.

## Materials & Methods

### Ethics approval statement

All experimental protocols have been approved by the Experimental Animal Management and Use Committee of Chifeng University, and all methods were carried out in accordance with approved guidelines and regulations (CFXY-IACUC-2025013).

### Animals and samples collection

Antler samples were collected from captive Dongbei red deer raised in a deer farm in Lindong Town, Balinzuo Banner, Inner Mongolia Autonomous Region, China. One healthy 4-year-old male Dongbei red deer was selected. After being injected with an anesthetic, three branches (designated as A1, A2, and A3) were harvested from the left antler branch that had been in the growth period for approximately 60 days. Immediately after cutting, hemostasis was performed, and the deer was subsequently revived. Next, the dermis and mesenchyme of antler samples were identified following the method described by Li et al. (2010). A medical surgical blade was used to cut the antler dermal tissue (dermis, D) and mesenchymal tissue (mesenchyme, M) into small pieces (approximately 0.5 cm in thickness). These tissue pieces were then (Li et al., 2002) immersed in RNA tissue preservation solution, frozen in liquid nitrogen, and stored for later use.

### RNA extraction from tissues and quality detection

Total RNA was extracted from liquid nitrogen-frozen tissues using TRIzol reagent, and the status of total RNA was detected by 1% agarose gel electrophoresis. Nanodrop-2100 was used to detect the concentration and purity of RNA, and Agilent 2100 was used to detect the integrity of RNA. The basic requirements for RNA quality to meet the needs of library construction were as follows: the concentration ≥ 100 (ng/μL), the total amount (μg) ≥ 1, and the volume (μL) ≥ 10. For the judgment of purity: the ratio of OD260/280 was between 1.7 and 2.5, and the ratio of OD260/230 was between 0.5 and 2.5. The requirement for the RNA Integrity Number (RNI) value was ≥ 7. After the samples passed inspection, the cDNA library was constructed.

### Construction and detection of the cDNA library

For the RNA samples that passed quality inspection, the conventional library construction method for lncRNA was used to construct the sequencing library. The steps were as follows: (1) removal of rRNA, (2) fragmentation of rRNA-depleted RNA, (3) synthesis of the first strand of cDNA, (4) synthesis of the second strand of cDNA and purification of the double-stranded products, (5) end repair and addition of an “A” at the 3′-end, (6) ligation of adapters, purification of the products, and PCR amplification, (7) purification of the PCR products, (8) quality inspection of the library.

### RNASeq and quality control and assembly of raw data

After passing the library quality inspection, high-throughput sequencing was performed on the dermal tissues (D group) and mesenchymal tissues (M group) with biological replicates M1, M2, M3, D1, D2, and D3 using the NovaSeq 6000 sequencing platform. Calculations were performed using the RNASeqPower package, with the following parameter settings: depth = 201, cv = 0.35, sample size = 3, effect = 2, and alpha = 0.05. The statistical power of this experimental design, calculated in RNASeqPower, is 0.67. The base calling analysis software used was Bcltofastq version 1.8.4, and the sequencing read length was 150 bp. Two technical replicates were performed for each sample to obtain raw data.

The sequencing library with confirm quality was loaded onto the sequencing machine for sequencing, and the original sequencing data were obtained using the Illumina NovaSeq 6000 sequencing platform. By removing the adapters and low-quality bases (Reads with the proportion of undetermined bases greater than 10% and Reads in which the number of bases with a base quality value (Q-score) ≤10 accounting for more than 50% of the entire Read, respectively) from the original data, high-quality Reads (Clean Reads) were obtained. The formula for base quality assessment was: Q-score = −10 ×log_10_ P. The specified genome Cervus_elaphus_hippelaphus was used as a reference for sequence alignment and subsequent analysis (Cervus_elaphus_hippelaphus.CerEla1.0.genome.fa). The Hierarchical Indexing for Spliced Alignment of Transcripts (HISAT) (Li et al., 2015) alignment system and the StringTie (Kim, Langmead, & Salzberg, 2015) software were used to assemble the Clean Reads to obtain transcripts. To ensure the sequencing quality, the quality of the transcriptome library was evaluated through the randomness test of mRNA fragments, the length distribution test of inserted fragments, and the gene saturation test.

### Calculation of gene expression levels and analysis of differentially expressed genes

The Bowtie (Langmead et al., 2009) software was used to align the sequenced Reads with the Unigene library. RNA-Seq by Expectation-Maximization (RSEM) (Li & Dewey, 2011) was used to estimate the expression level, and the Fragments Per Kilobase of transcript per Million mapped reads (FPKM) (Trapnell et al., 2010) value was used to represent the expression level of the corresponding Unigenes. The skin tissue was set as the control group. Differential Expression analysis based on negative binomial distribution (DESeq) (Love, Huber & Anders, 2014) was used to compare the differences in gene expression levels between skin tissue and mesenchymal tissue, and a set of differentially expressed genes (DEG) was obtained. The false discovery rate (FDR) and the fold change (FC) were used as the key indicators for screening differentially expressed genes. The screening criteria were set as FDR <0.01 and FC ≥ 2.

### Functional annotation and enrichment analysis of differentially expressed genes

The differentially expressed gene sequences obtained through screening were compared with bioinformatics databases including COG, KOG, GO, KEGG, Swiss-prot, eggNOG, NR, and Pfam using Blastx to obtain the annotation information of the genes. The Blast parameter was set as *E*-value ≤ 1e−5. The differentially expressed genes were classified according to the three basic functional categories in the GO database, namely “cellular component” (CC), “molecular function” (MF), and “biological process” (BP) to obtain the biological function annotations related to the functions of the differentially expressed genes. Functional annotation in the KEGG database was carried out to obtain the pathways of the differentially expressed genes and to interpret the functions of the genes. The GO functions and KEGG pathway enrichment of the differentially expressed genes among samples were performed, and the different trends of enrichment of all genes were analyzed to annotate the functions of the genes. The ClusterProfiler (Yu et al., 2012) was used to perform enrichment analysis of the biological processes, molecular functions, and cellular components of the genes. The hypergeometric test method was adopted to find significantly enriched GO terms and KEGG pathways.

### Quantitative verification of differentially expressed genes

To confirm the accuracy of the gene expression levels in the transcriptome, four genes were verified using the real-time fluorescence quantitative quantitative reverse transcription polymerase chain reaction (qRT-PCR) method, with β-actin as the internal reference gene. The total RNA extraction method was the same as described above. Approximately twoµg total RNA was reverse transcribed using the TRUEscript 1st Strand cDNA Synthesis Kit (PC1802, Aidlab Biotechnologies Co., Ltd.). Complete reaction conditions: two µg total RNA, 0.5 μL Oligo (dT) (10 μM), 0.5 μL Random primer (N6) (10 μM), 4 μL 5 × TRUE Reaction Mix, 0.8 μL TUREscript H- RTase/RI Mix (200 U/μL), RNase-free water up to 20 μL. Reactions were incubated at 42 °C for 40 min, then at 65 °C for 11 min to inactivate enzyme, and cDNA was then stored at −80 °C. The Primer 5.0 software was used to design the primers for the corresponding genes according to the sequences obtained from the transcriptome of antlers (Table 1). In silico specificity screening using BLAST showed that the primers specifically bound to the target gene with no significant homology to other genes in the genome. Primer DNA and Oligo(dT) were manufactured by Tsingke (Beijing Tsingke Biotech Co., Ltd.). Primers were purified by PAGE, 10 μL. The qRT-PCR assay was performed using HotMaster Taq DNA polymerase (included in Aidlab Biotechnologies Co., ltd) and BIO-RAD CFX Connect Real-Time System. The robotic setup used q-PCR Fully automatic Sampler (Model M1, Sichuan KeJin, Chengdu, China) liquid handler. The reaction system was as follows: 1μL cDNA (40 ng), 5 μL 2 × SYBR Green Master Mix (Aidlab Biotechnologies Co., Ltd., PC3302), 0.5 μL forward primer (10 μM), 0.5 μL reverse primer (10 μM), and 3 μL RNase-free water. The reaction condition was as follows: pre - denaturation: 95 °C for 3 min; amplification (40 cycles): 95 °C for 10 s, 60 °C for 30 s; the melting curve was: 95 °C for 10 s, 60 °C for 10s, then ramp to 95 °C at 0.5 °C/s.

**Table 1:** The sequences of the primers for fluorescence quantitative genes.

Gene name	Primers
*ACT*-F	GCGTGACATCAAGGAGAAGC
*ACT*-R	GGAAGGACGGCTGGAAGA
*CASP3*-F	CCTGAAGAACAAGTCCTGAAT
*CASP3*-R	CTGCCAAGAGTGCTATGATT
*MDM2*-F	AAGAAGAAAGCGTGGAGTC
*MDM2-*R	ATTTGAATGGGTTGCCTACA
*BMP4*-F	TGGAACGACTGGATTGTG
*BMP4*-R	ATGGTTGGTGGAGTTGAG
*PTCH1*-F	GTTGGTGTGGATGATGTCT
*PTCH1*-R	GTTGCTGATGGAGGTGAG

The QPCR analysis program Bio-Rad CFX Maestro 2.2 (Bio-Rad) was applied. Ct values were determined by automatic threshold setting in Applied Bio-Rad CFX Maestro 2.2 and were placed in the exponential phase of amplification curves. Data normalization was performed using the 2^−ΔΔCt^ method. Three technical replicates were performed at the qPCR stage for each sample. Statistical significance of differences in gene expression was determined using a two-tailed Student’s *t*-test for pairwise comparisons. Statistical analysis was performed using GraphPad Prism. Relative gene expression was calculated using the comparative 2^−ΔΔCt^ method.

## Results

### Sample collection and total RNA extraction

The concentrations of RNA extracted from the dermal tissue and mesenchymal tissue of the Dongbei Red Deer antlers were greater than 550.2 and 908.6 ng μl^−1^, respectively. The RIN values of the dermal and mesenchymal tissue were greater than 7 and 9, respectively, the OD_260_/_280_ ratio was greater than 2, the 28S/18S ratio was greater than 2.7, and the integrity of the RNA purity met the requirements for library construction.The datasets are available in NCBI SRA BioProject: PRJNA1268152.

### Quality control and assembly of sequencing data for skin and mesenchymal tissues

Transcriptome sequencing of six samples of dermal layer (D1, D2 ,D3) and mesenchymal layer (M1, M2, M3) tissues was completed, and a total of 109.82 Gb of Clean Data was obtained. The Clean Data of each sample reached 16.73 Gb. The percentage of Q30 bases was above 92.78%, and the average Guanine-Cytosine (GC) content was 50.73%. The Clean Reads of each sample were aligned with the genome of Cervus elaphus. The alignment efficiency ranged from 78.74% to 81.94% (Table 2), all of which were higher than 70%. The aligned reads were assembled using StringTie (Kim, Langmead & Salzberg, 2015), and a total of 40,023 transcripts were obtained, including 5,935 new genes.

**Table 2:** The comparison results of sample sequencing data.

**Samples**	**Total reads**	**Mapped reads**	**Uniq mapped reads**
D1	133,718,918	107,204,501 (80.17%)	104,356,876 (78.04%)
D2	127,587,744	101,453,842 (79.52%)	98,378,444 (77.11%)
D3	111,964,762	88,161,526 (78.74%)	86,117,495 (76.91%)
M1	119,172,088	95,820,456 (80.41%)	92,568,727 (77.68%)
M2	116,808,676	95,718,387 (81.94%)	91,976,562 (78.74%)
M3	125,908,844	101,669,556 (80.75%)	98,472,387 (78.21%)

### Analysis of the correlation coefficient of samples

**Figure 1:**
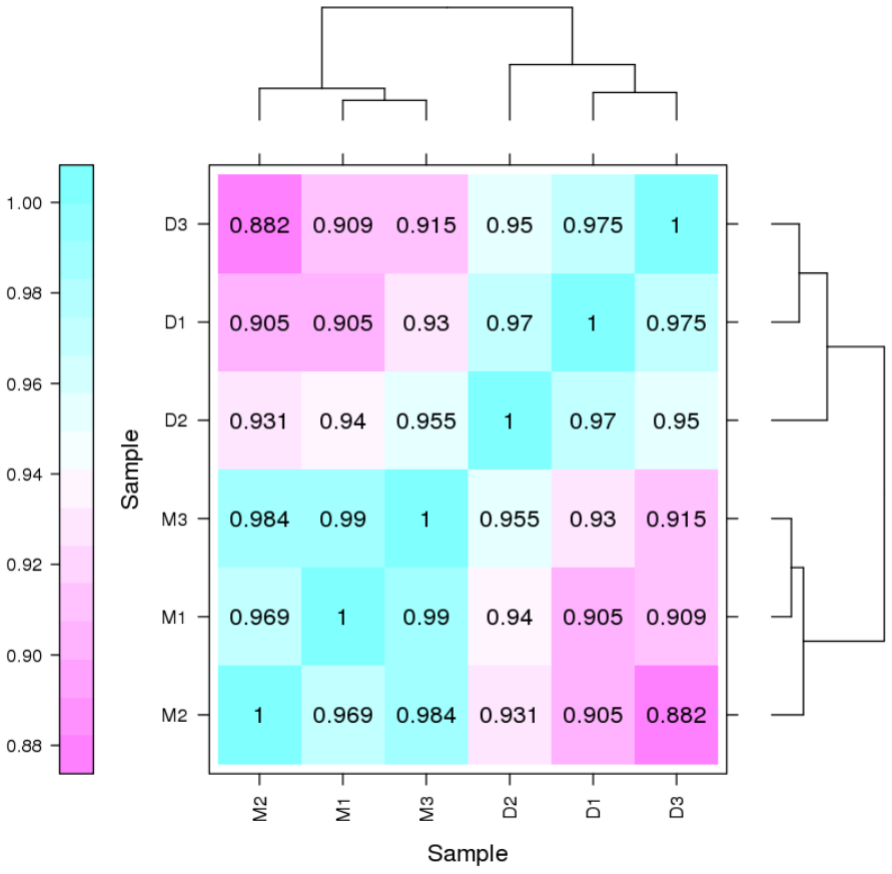
Correlation analysis of samples.

The Pearson’s correlation coefficient r was used as the evaluation index for the correlation of biological replicates. The closer r^2^ is to 1, the stronger the correlation between the two replicate samples. Generally, an r^2^ is greater than 0.9 is considered to be a good correlation. The correlation coefficients of the six samples are shown in Fig. 1. It can be seen that the correlation coefficients between each sample are all greater than or equal to 0.88, and the correlation coefficients between the samples within the group are greater than those between the samples outside the group.

### Analysis of gene differential expression and functional annotation

Using the dermis tissue as a control to calculate the gene expression levels, 3,530 differentially expressed genes were obtained. Among them, 205 genes were specifically expressed in the skin, and 13 genes were specifically expressed in the mesenchyme. There were 2,378 genes with upregulated expression levels and 1,153 genes with downregulated expression levels. By comparing the gene sequences with the bioinformatics databases of COG, KOG, GO, KEGG, Swiss-prot, eggNOG, NR, and Pfam through Blastx, functional annotations were obtained for 3,038 genes.

### Gene Ontology (GO) functional enrichment of differentially expressed genes

The results of the GO functional enrichment analysis of differentially expressed genes showed that 2,777 genes were enriched in 57 subcategories within three major categories (biological processes, cellular components, and molecular functions). The results of the enrichment of differentially expressed genes showed that the biological processes in which these genes were mainly enriched were cell adhesion, signal transduction, epidermal development, cell surface receptor signaling pathways, and lipid metabolic processes. The cellular components in which they were mainly enriched were keratin filaments, intrinsic components of the cell membrane, intermediate filaments, extracellular region part, and extracellular region. The molecular functions in which they were mainly enriched were calcium ion binding, guanine nucleotide exchange factor activity, structural components of the extracellular matrix for tensile strength, structural molecular activity, and semaphorin receptor binding (Table 3). An enrichment analysis was conducted on the genes with upregulated expression in the mesenchymal layer. The biological process in which the upregulated genes were most enriched was the metabolism of phosphate compounds. In addition, the genes were also enriched in multiple biological processes related to bone development such as skeletal system development, cartilage development, regulation of ossification, and enamel mineralization. The primarily enriched cellular components were the cytoplasm and the extracellular region, and the mainly enriched molecular functions were insulin receptor binding, growth factor binding, and calcium ion binding (Figs. S1, S2, S3).

**Table 3:** The GO functions in which the differentially expressed genes are primarily enriched.

Function categories	GO Term	Gene ratio	Enrich factor	*p* value	*q* value
Biological processes	Cell adhesion	4.39%	1.83	3.01E−07	0.00140
	Signal transduction	11.95%	1.39	5.97E−07	0.00140
	Epidermis development	1.46%	2.8	1.88E−06	0.00227
	Cell surface receptor signaling pathway	5.40%	1.63	2.30E−06	0.00227
	Lipid metabolic process	3.56%	1.85	2.42E−06	0.00227
Cellular components	Keratin filament	1.11%	4.84	7.82E−06	0.00612
	Integral component of membrane	40.95%	1.2	1.73E−05	0.01141
	Intermediate filament	1.28%	3.75	2.21E−05	0.01141
	Extracellular region part	3.88%	1.79	2.30E−05	0.01141
	Extracellular region	8.55%	1.44	2.52E−05	0.01141
Molecular functions	Calcium ion binding	6.70%	1.61	5.54E−11	0.00000
	Guanyl-nucleotide exchange factor activity	3.09%	1.87	6.98E−11	0.00000
	Extracellular matrix structural constituent conferring tensile strength	0.34%	6.48	2.44E−09	0.00000
	Structural molecule activity	2.75%	1.72	5.34E−07	0.00009
	Semaphorin receptor binding	0.58%	3.56	8.33E−07	0.00011

### KEGG functional enrichment of differentially expressed genes

The results of KEGG annotation analysis of differentially expressed genes showed that 1,498 differentially expressed genes were enriched in six major categories, including cellular processes, environmental information processing, metabolism, genetic information processing, organismal systems, and human diseases, across 287 pathways. Among them, the top 20 pathways with a relatively large number of differentially expressed genes were the pathways in cancer, the PI3K-Akt signaling pathway, focal adhesion, and axon guidance (Table 4). The genes with upregulated expression levels were enriched in *Staphylococcus aureus* infection, extracellular matrix-receptor interaction, cancer-related pathways, axon guidance, and the PI3K-Akt signaling pathway.

**Table 4:** The top 20 pathways of KEGG enrichment of differentially expressed genes.

KEGG pathways	Gene ratio	Enrich factor	*p* value	*q* value
Pathways in cancer	10.08%	1.49	9.30E−08	5.69E−06
PI3K-Akt signaling pathway	7.14%	1.59	3.75E−07	1.70E−05
Human papillomavirus infection	5.61%	1.32	3.75E−03	2.07E−02
Axon guidance	4.74%	1.84	1.01E−07	5.69E−06
Focal adhesion	4.74%	1.55	7.85E−05	1.27E−03
MAPK signaling pathway	4.67%	1.23	3.59E−02	8.05E−02
Proteoglycans in cancer	4.21%	1.54	2.36E−04	2.54E−03
Cytokine-cytokine receptor interaction	4.14%	1.55	2.26E−04	2.54E−03
Ras signaling pathway	4.07%	1.42	2.44E−03	1.53E−02
Calcium signaling pathway	4.01%	1.26	3.33E−02	7.85E−02
Rap1 signaling pathway	3.94%	1.36	7.54E−03	3.07E−02
ECM-receptor interaction	3.87%	2.29	3.05E−10	3.45E−08
Protein processing in endoplasmic reticulum	3.74%	1.7	2.88E−05	5.44E−04
Endocytosis	3.74%	1.09	2.65E−01	3.38E−01
Human cytomegalovirus infection	3.67%	1.41	4.67E−03	2.40E−02
Regulation of actin cytoskeleton	3.54%	1.14	1.69E−01	2.52E−01
cAMP signaling pathway	3.54%	1.05	3.79E−01	4.40E−01
Herpes simplex virus 1 infection	3.54%	0.57	1.00E+00	6.94E−01
Wnt signaling pathway	3.27%	1.42	6.49E−03	2.83E−02
Cell adhesion molecules	3.27%	1.4	7.63E−03	3.07E−02

### Screening of differentially expressed genes

Combined with the results of GO enrichment and KEGG signaling pathway enrichment analysis of differentially expressed genes, genes related to the growth and development functions of antlers were screened. It was found that there were 205 genes specifically expressed in the dermal layer and 13 genes specifically expressed in the mesenchymal layer. Among these genes, the epithelial-specific ETS homologous factor (*EHF*) gene was expressed. In addition, six keratin genes (*KRT44*, *KRT6A*, *KRT81*, *KRT34*, *KRT23*, *KRT24*) as well as the bone morphogenetic protein 3 gene (*BMP3*) and hepatocyte growth factor gene (*HGF*) were specifically expressed in the dermal layer. Genes including type I collagen α2 chain (*COL1A2*), sodium-calcium exchanger (*SLC8A1*), calmodulin (*CLGN*), hedgehog protein (*IHH*), and transcription factor homeobox protein (*HOXB4*) were specifically expressed in the mesenchymal layer. Among them, *COL1A2* is abundant in most connective tissues. Sodium-calcium exchanger and calmodulin play roles in calcium ion binding and transportation during cell growth and development. Hedgehog protein and transcription factor homeobox protein are important signal transduction proteins.

There were some genes with differential expressions related to cell activities such as cell proliferation and differentiation, cell cycle regulation, and apoptosis that were identified in the cancer pathway and the PI3K-Akt pathway. Eight genes (Table 5) with upregulated expression levels were screened out, and their main functions include promoting cell proliferation, inhibiting apoptosis, and promoting the cell cycle process.

**Table 5:** Screening of genes with upregulated expression levels in the mesenchymal tissue.

KEGG pathways	**Gene name**	**M *vs* D**
Pathway in cancer	E3 ubiquitin-protein ligase (*MDM2*)	1.1032
	ribosomal protein S6 kinase beta-1 (*RPS6KB1*)	1.2567
	caspase-3 (*CASP3*)	1.3106
	bone morphogenetic protein 4 (*BMP4*)	1.9948
	protein patched homolog 1 (*PTCH1*)	2.8991
	transcription factor SOX-6 (*SOX6*)	3.5961
PI3K-Akt signaling pathway	14-3-3 protein zeta (*YWHAZ*)	1.0712
	endoplasmin (*HSP90B1*)	1.2019

### Fluorescent quantitative analysis of gene expression levels

**Figure 2:**
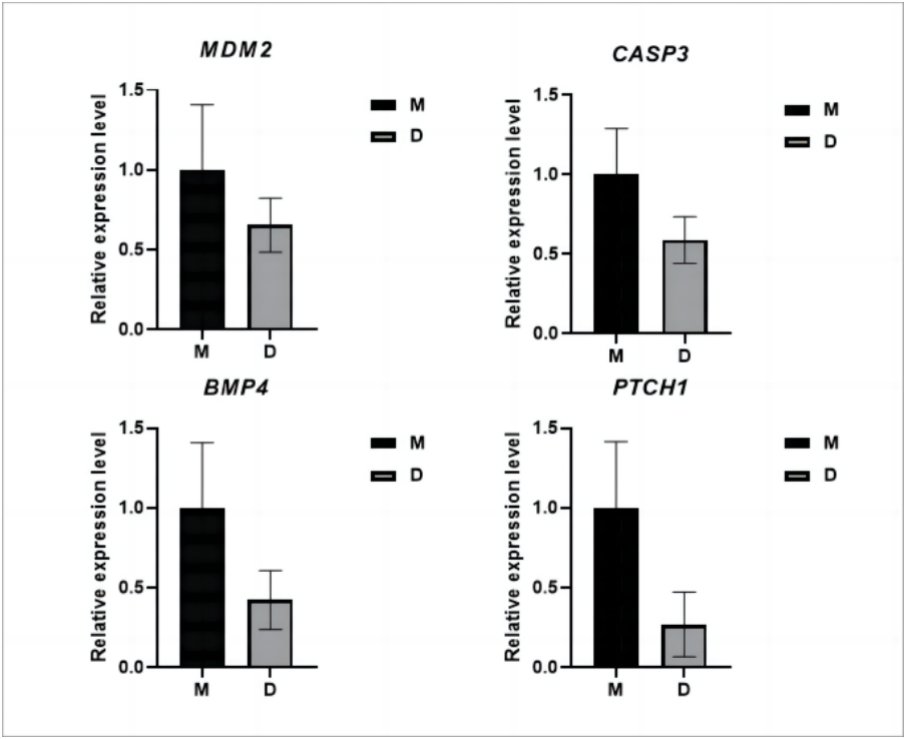
RT-qPCR verification of differentially expressed genes.

Using the *ACT* gene as an internal reference, the genes of *CASP3*, *MDM2*, *BMP4*, and *PTCH1* were selected for the fluorescence quantitative experiment. The results showed that the expression trends of the transcriptome sequencing results of these four upregulated genes were consistent with those of the qRT-PCR detection results (Fig. 2).

## Discussion

### Genes with tissue-specific expression in different structures of deer velvet antler

This study performed transcriptome sequencing on the dermal and mesenchymal layers of antler velvet from the Dongbei red deer. In the dermal layer, the ETS homolog factor (EHF) gene was identified, which is specifically expressed in differentiated epithelial tissues (Ma & Guo, 2023; Cheng et al., 2016), as well as six keratin genes *(KPT44*, *KPT6A*, *KPT81*, *KPT34*, *KPT23*, *KPT24*) that constitute skin tissue. In the mesenchymal layer, there was specific expression of the *COL1A2* gene, encoding type I collagen chains, along with the sodium-calcium exchanger gene (*SLC8A1*) and the calmodulin gene (*CLGN*). The tissue-specific expression of these genes in the dermal and mesenchymal layers clearly highlights the heterogeneity of cellular composition between these two tissue layers. Notably, this study revealed that type I collagen is specifically expressed in the mesenchymal layer. Type I collagen is a major component of skin, bone, and cornea (Han, 2020). The *COL1A2* gene, currently detected in various cancer tissues and sika deer antler velvet (Zhang et al., 2018), is hypothesized to be associated with cancer development (Yang et al., 2024) and may also be linked to the rapid, non-cancerous growth characteristic of antler tissue. The sodium-calcium exchanger (SLC8A1) and calmodulin (CLGN) play crucial roles in calcium ion binding and transport. The tissue-specific expression of genes involved in calcium ion binding and transport suggests a close relationship between the formation of antler cartilage and the regulation of calcium ion uptake and transport. In addition, the dermal layer exhibits specific expression of two growth factors: bone morphogenetic protein 3 (BMP3) and hepatocyte growth factor (HGF). BMP3 regulates the differentiation of osteoblasts and chondrocytes during skeletal development. HGF, upon binding to its receptor, is widely distributed in various tissue cells and modulates cell growth and differentiation of the occurrence of tissue morphology. The phenomenon that these two proteins are specifically expressed in the dermis tissue suggests that the development of the skin tissue may play a regulatory role in the development of the antler (Han et al., 2020).

### Differential expression of factors related to cancer pathways and the PI3K-AKT pathway in different tissue structures of deer velvet antler

Functional enrichment analysis was performed on genes differentially expressed in the dermal and the mesenchymal layer. The results showed that among the six genes upregulated in the mesenchymal layer and associated with cancer pathways, several are involved in regulating cell apoptosis, cell proliferation, growth, and differentiation. These include E3 ubiquitin-protein ligase (MDM2), ribosomal protein S6 kinase beta-1 (RPS6KB1), caspase-3 (CASP3), bone morphogenetic protein 4 (BMP4), and patched homolog 1 (PTCH1). Existing data have demonstrated that the products of these genes play crucial roles in regulating tumor formation and the progression of cancer cell development. For instance, E3 ubiquitin-protein ligase (MDM2) inhibits normal programmed cell death by ubiquitinating the p53 protein, thereby contributing to the tumorigenesis process (Hua, 2003; Wang & Li, 2021). Consequently, the MDM2 gene is also referred to as an oncogene (Guo, 1994). Ribosomal protein S6 kinase beta-1 (RPS6KB1) exhibits abnormal expression patterns in various malignant tumors and exerts effects on regulating tumor cell growth, proliferation, cell cycle progression, and apoptosis pathways, thus promoting cell survival (Yang, 2023). Caspase-3 (CASP3), a member of the cysteine protease family, is closely associated with eukaryotic cell apoptosis and participates in the regulation of cell growth, differentiation, and apoptosis; it may also exert an inhibitory effect on tumors (Zhou et al., 2018). Bone morphogenetic protein 4 (BMP4), a growth factor belonging to the TGF-*β* superfamily, plays a critical role in developmental processes such as neurogenesis, angiogenesis, vasculogenesis, and osteogenesis. Studies have shown that mutations in BMP4 can affect bone mass in teeth, highlighting the protein’s essential function in skeletal formation (Yu et al., 2019). Patched homolog 1 (PTCH1) serves as the receptor for hedgehog proteins and binds to smoothened (SMO) to transduce hedgehog signaling. This protein is implicated in tumor initiation and progression.

The PI3K-AKT pathway is an intracellular signaling cascade that responds to extracellular signals and promotes metabolism, proliferation, cell survival, and growth. The genes encoding the 14-3-3 protein zeta (YWHAZ) and the heat shock protein gene (HSP90B1), which are involved in this signaling pathway, show increased expression levels in the mesenchymal layer. The 14-3-3 protein zeta belongs to the 14-3-3 protein family and serves as a central hub protein in many signaling pathways, playing a critical role in tumor progression. Studies have shown that YWHAZ is frequently upregulated in various types of cancer and acts as an oncogene in a wide range of cellular processes, regulating cell growth, apoptosis, and other cellular activities (Gan, Ye & He, 2020). The member of the heat shock protein 90 beta family (HSP90B1) exhibits significantly elevated expression levels in many tumor cells. HSP90B1 helps stabilize the folding and activation of oncoproteins in tumor cells, thereby promoting tumor cell proliferation, survival, invasion, and metastasis. Numerous studies have also highlighted the close correlation between the expression and function of 14-3-3 proteins and HSP90 proteins (Huangfu & Fuchs, 2010; Fristrup et al., 2013; Kim & Kim, 2012; Sui et al., 2018; Ren et al., 2023; Dewson, Eichhorn & Komander, 2023). Specifically, 14-3-3*ɛ* activates the AKT/mTOR pathway by upregulating HSP90B1 expression, suggesting that HSP90B1 may play a role in biological processes mediated by 14-3-3*ɛ* (Shi, 2024). Further in-depth research is needed to elucidate the roles of these genes—expressed during the rapid growth of deer antlers and driving cancer cell proliferation and tumor growth—as well as the roles of 14-3-3*ɛ* protein and the HSP90B1 gene in regulating and controlling deer antler growth.

The expression of sex-determining region Y-box protein 6 (SOX-6) is upregulated in the mesenchymal layer. SOX-6 is a member of the SOXD family, which is widely involved in mesenchymal tissue differentiation, central nervous system development, and promotion of erythropoiesis (Cao et al., 2021). Recent studies have shown that SOX-6 influences cartilage formation by specifically binding to particular DNA motifs during the differentiation of condensed prechondrocytes into early-stage chondrocytes. The ossification mechanism of deer antlers involves endochondral ossification, during which a stage exists characterized by the differentiation of mesenchymal cells into chondrocytes and the formation of prechondral tissue. The role of the transcription factor SOX-6 in this process requires further investigation.

### The significance of genes highly expressed in the mesenchyme of rapidly growing antler

We identified, for the first time, eight genes significantly upregulated (FC ≥ 2, FDR < 0.01) in the mesenchyme layer of 60-day-old antler from Cervus elaphus xanthopygus (Dongbei red deer). These genes are enriched in cancer and PI3K-AKT signaling pathways: *MDM2*, *RPS6KB1*, *CASP3*, *BMP4*, *PTCH1*, *SOX6*, *YWHAZ*, and *HSP90B1*. qPCR validation confirmed that their expression trends were consistent with the transcriptomic data. This study represents the first identification of these eight candidate genes at the RNA level within the antler velvet mesenchyme that are potentially associated with its rapid growth.

### Limitations of the current research methodology and future directions

(1) Sample size: A single individual only allows for the detection of differential expression but fails to account for biological variation among individuals; therefore, subsequent studies will require a sample size of n ≥ 5 male individuals.

(2) Time points: While 60 days corresponds to the peak growth phase, the absence of earlier (30 d) and later (90 d) time points limits the ability to resolve the temporal dynamics of gene expression.

(3) Causality: Current findings provide only potential correlative evidence. To establish a mechanistic link, it is necessary to perform CRISPR-mediated knockout or overexpression in primary antler mesenchymal cells and observe the resulting phenotypes regarding proliferation and chondrogenic differentiation.

(4) Tissue purity: This study relied on anatomical dissection for layering without histological quantification. Future research should incorporate fluorescent labeling (*e.g.*, COL1A2-GFP) combined with single-cell RNA sequencing (scRNA-seq) to exclude interference from cellular heterogeneity.

## Conclusion

This study identified eight genes potentially associated with rapid growth in the mesenchyme layer of 60-day-old Cervus elaphus xanthopygus antler: *MDM2*, *RPS6KB1*, *CASP3*, *BMP4, PTCH1*, *SOX6*, *YWHAZ*, and *HSP90B1*. Future studies will require expanded sample sizes, additional time-series data, and functional interference experiments to validate the biological functions of these genes.

## Supplemental Information

10.7717/peerj.21606/supp-1 Supplemental Information 1 MIQE Checklist Detection of mRNA by quantitative Real-time PCR

10.7717/peerj.21606/supp-2 Supplemental Information 2 Biological process terms enriched in up-regulated expressed genes

10.7717/peerj.21606/supp-3 Supplemental Information 3 Cellular component terms enriched in up-regulated expressed genes

10.7717/peerj.21606/supp-4 Supplemental Information 4 Molecular function terms enriched in up-regulated expressed genes

10.7717/peerj.21606/supp-5 Supplemental Information 5 ARRIVE Checklist

10.7717/peerj.21606/supp-6 Supplemental Information 6 Detection report of mRNA by quantitative Real-time PCR

## Additional Information and Declarations

### Data Availability

The following information was supplied regarding data availability: The raw sequence data are available at NCBI BioProject: PRJNA1268152.
