# Supplementary figures and images for: Transcriptomic characterization of mesenchymal and skin tissues in Cervus elaphusxanthopygus antler and identification of growth-related candidate genes
Source: PeerJ. 2026 Jul 27;14:e21606. doi: 10.7717/peerj.21606 (PMC13421806; doi:10.7717/peerj.21606)

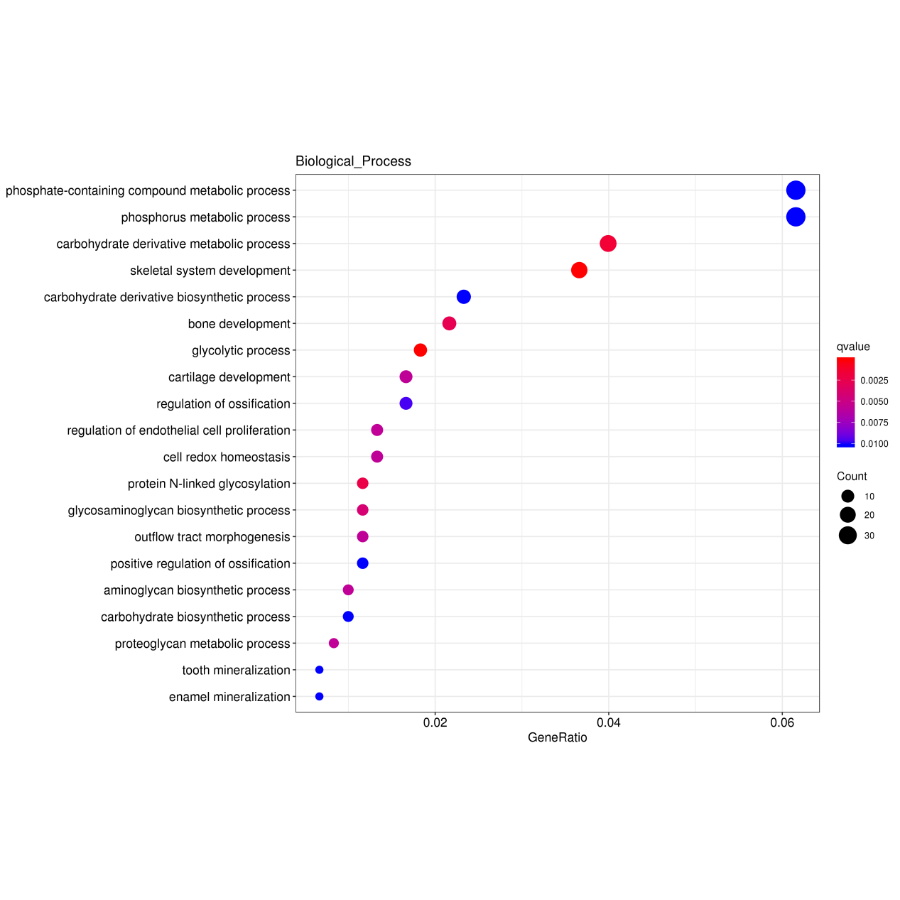

Supplement: Supplemental Information 2 [file peerj-14-21606-s002.png]

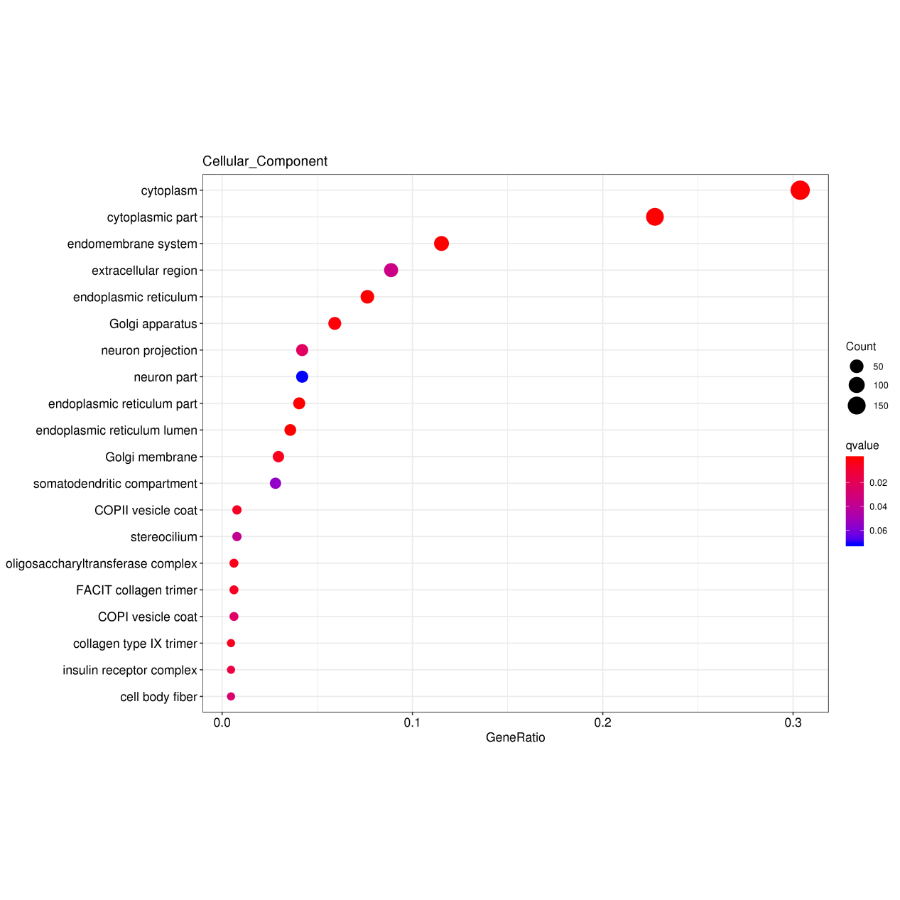

Supplement: Supplemental Information 3 [file peerj-14-21606-s003.png]

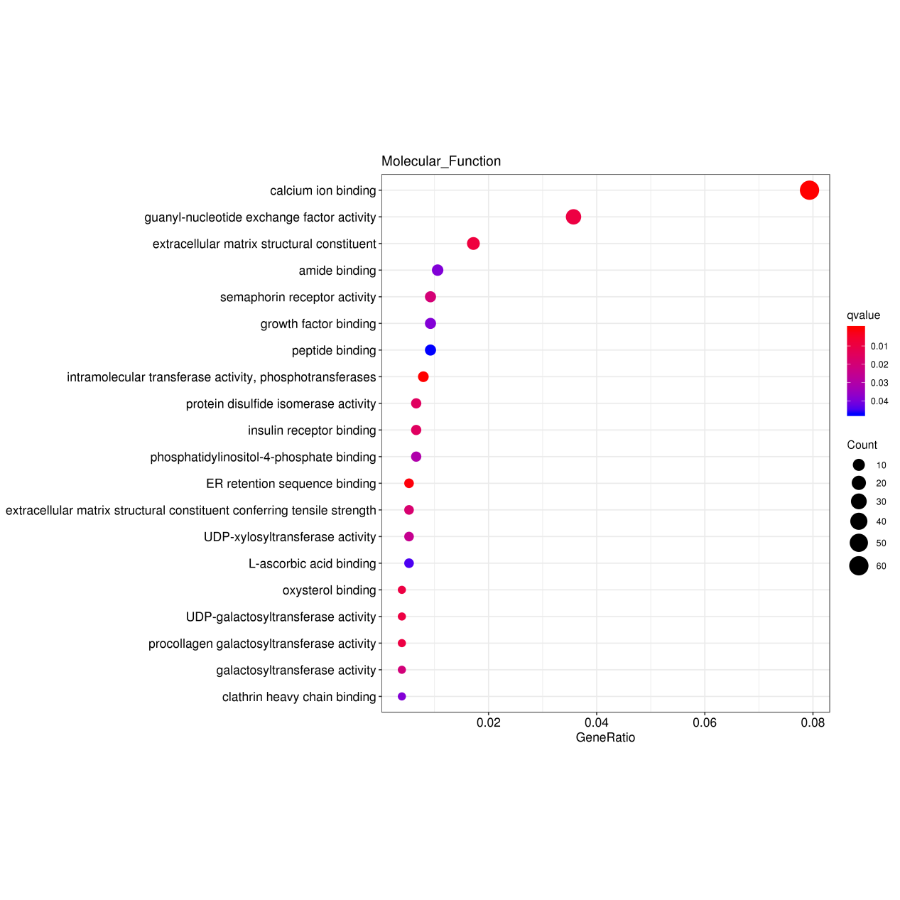

Supplement: Supplemental Information 4 [file peerj-14-21606-s004.png]
