# Supplementary material for: Transcriptomic characterization of mesenchymal and skin tissues in Cervus elaphusxanthopygus antler and identification of growth-related candidate genes
Source: PeerJ. 2026 Jul 27;14:e21606. doi: 10.7717/peerj.21606 (PMC13421806; doi:10.7717/peerj.21606)
Supplement: Supplemental Information 6 [file peerj-14-21606-s006.pdf]

Detection report of mRNA by quantitative Real-time PCR

1 Material

2 groups antler tissue samples named LRM (LRM1、LRM2、LRM3) and MLD (MLD1、MLD2、MLD3) was prepared for total RNA extraction.

2 Equipment and Reagents

2.1 Equipment

qTOWER 2.0/2.2 Quantitative Real-Time PCR Thermal Cyclers(Germany), Analytik Jena(Germany). SCILOGEX D3024R High Speed Refrigerated Micro-Centrifuge(USA). Scandrop100. Pipette: 10μL, 100μL, 1000μL(Bio-rad)，Automatic sample adding instrument-M1（SiChuanKeJing）.

2.2 Main Reagents and consumables

TUREscript 1st Stand cDNA SYNTHESIS Kit(Aidlab). 2×SYBR® Green Master Mix(DBI). Pipet tips: 10μL, 100μL, 1000μL (GCS). 0.2ml microcentrifuge tube (RNase, AXGEN). 1.5ml centrifuge tube (RNase, GCS). Multiplate™ 96-well unskirted PCR plate. Microseal® 'B'adhesive seals. Pipet tips and centrifuge tube were sterilized and dried before use.

3 Experimental method

3.1 Total RNA extraction from antler tissues samples

Add 1000μl Trizol to the antler tissuse samples,respectively. Fully mixed and incubate at room temp for 30 minutes. Then add 200μl chloroform, fully vortex and incubate at room temp for 10 minutes. Centrifuge samples at 12,000g for 15 minutes at 2-8°C. After contrifugation, transfer 400μl supernatant liquid to a fresh clean tube (1.5ml), and add 500μl isopropanol. Upside down of several times and incubate at 4°C overnight. Centrifuge samples at 12,000g for 10 minutes at 2-8°C and discard the supernatant liquid. Add 1000μl 75% alcohol and centrifuge at 7,500g for 5 minutes at 4°C. Discard the alcohol and add 30μl ddH_2O to dissolve the RNA after all alcohol volatilized. Keep the RNA in ice for 15-20 minutes and then quantify each RNA sample by scandrop 100.

Table 1 RNA quality results

No.	Sample name	Absorbance A260/A280	Concentration ng/μL
1	LRM1	1.88	548.3
2	LRM2	1.93	734.5
3	LRM3	1.95	642.3
4	MLD1	1.91	748.8
5	MLD2	1.82	1024
6	MLD3	1.92	2330

3.2 First strand cDNA systhesis

Operated as the handbook of TUREscript 1st Stand cDNA SYNTHESIS Kit to systhesis cDNA. Choose 20μl reaction system or what volume you need.

Total RNA	1000ng
5×RT Reaction Mix	4uL
Rondam primer/oligodT	1uL
TUREscript H^- RTase/RI Mix	1uL
RNase Free dH_2O	to 20ul

Reaction condition

42℃	40min
65℃	10min

The cDNA should be kept in -20℃.

3.3 Primer design and synthesis

Primers were disigned by Beacon Designer 7.9.

Table 2 Prime information

	Primers	5'to3'	TM
	Actin-F	GCGTGACATCAAGGAGAAGC	60
	Actin-R	GGAAGGACGGCTGGAAGA	
1	CASP3-F	CCTGAAGAACAAGTCCTGAAT	60
	CASP3-R	CTGCCAAGAGTGCTATGATT	
2	MDM2-F	AAGAAGAAAGCGTGGAGTC	60
	MDM2-R	ATTTGAATGGGTTGCCTACA	

3	BMP4-F	TGGAACGACTGGATTGTG	60
	BMP4-R	ATGGTTGGTGGAGTTGAG	
4	PTCH1-F	GTTGGTGTGGATGATGTCT	60
	PTCH1-R	GTTGCTGATGGAGGTGAG	

3.4 quantitative Real-time PCR

Reaction condition

Step1- 95 ℃-3 Min

Step2- 95 ℃-10 s

Step3- 58 ℃-30 s +plate read

Step5- Go to step2, 39 cycles

Step6- Melt curve analysis（60 ℃~ 95 ℃，+1 ℃/cycle，holding time 4 s）。

After add all components, centrifuge at 6,000rpm for 1 minutes to keep all components in the bottom.

Table 3 qPCR components and dosage

Components	Final concentration	Loading volume (μL)
2×SYBR® Green Supermix	1×	5
Reverse primer	200nM	0.5
Sense primer	200nM	0.5
cDNA	N/A	1
ddH$_2$O	N/A	3
Total		10

4 Result

4.1 Detection result

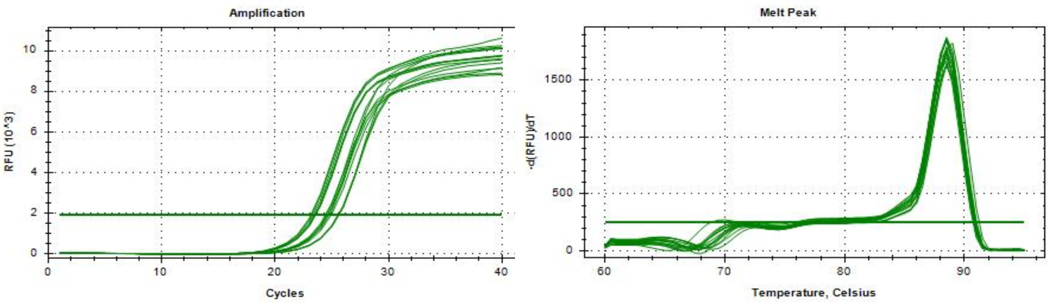

Fig.4.1 Internal control gene: Actin

Left: Amplification curve, Right: melting curve, TM 82℃

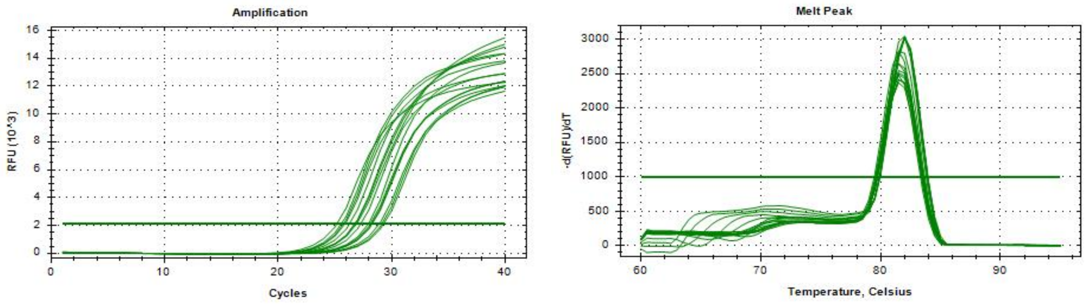

Fig.4.2 Target gene: MALAT1

Left: Amplification curve, Right: melting curve, TM 81℃

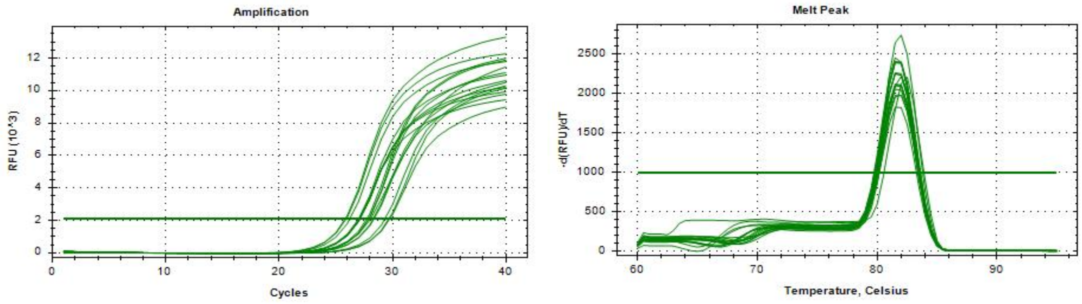

Fig.4.3 Target gene:DNCR

Left: Amplification curve, Right: melting curve, TM 77℃

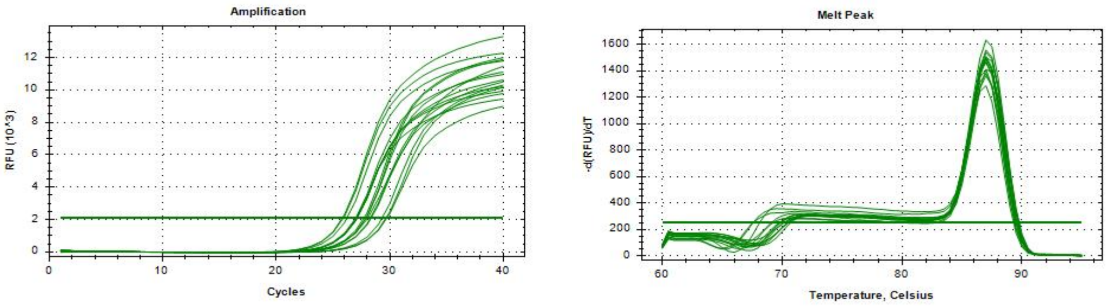

Fig.4.4 Target gene:TINCR

Left: Amplification curve, Right: melting curve, TM 77℃

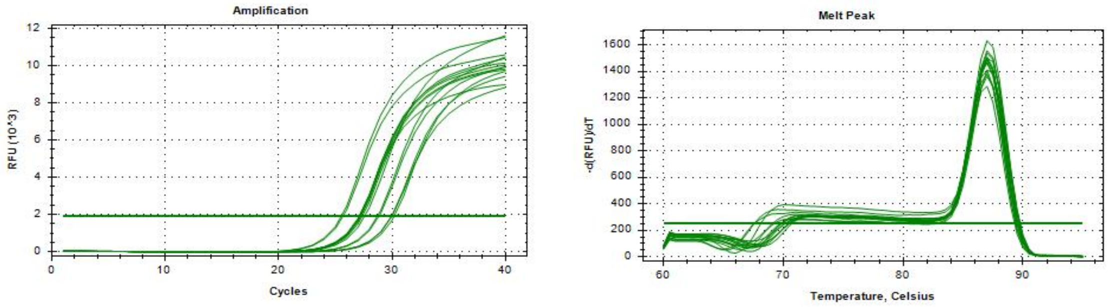

Fig.4.5 Target gene:RUNX2

Left: Amplification curve, Right: melting curve, TM 77℃

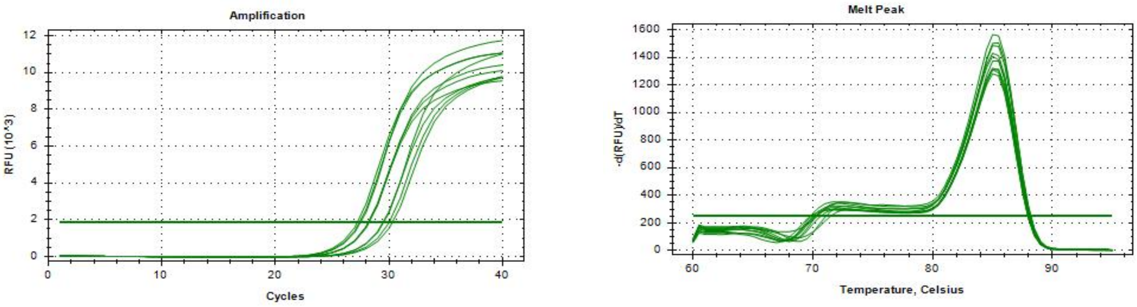

Fig.4.6　Target gene:RUNX2

Left: Amplification curve, Right: melting curve,　TM 77℃

4.2 Analysis

Set 3 technical repetition for each sample. The relative gene expression can be calculated by the qPCRsoft3.2 software automatically. Pfaffl method were used by the software and the formule were:

$$\text{Ratio} = \frac{(1+E_{\text{target}})^{\Delta \text{Ct target (control}-\text{expt)}}}{(1+E_{\text{reference}})^{\Delta \text{Ct reference(control}-\text{expt)}}}$$

3 Conclude

Calculated the relative expression, one of the samples were set as control group and gene Actin were set as internal control. The relative gene expression 2^-△△Ct were turned into graphs as follows.

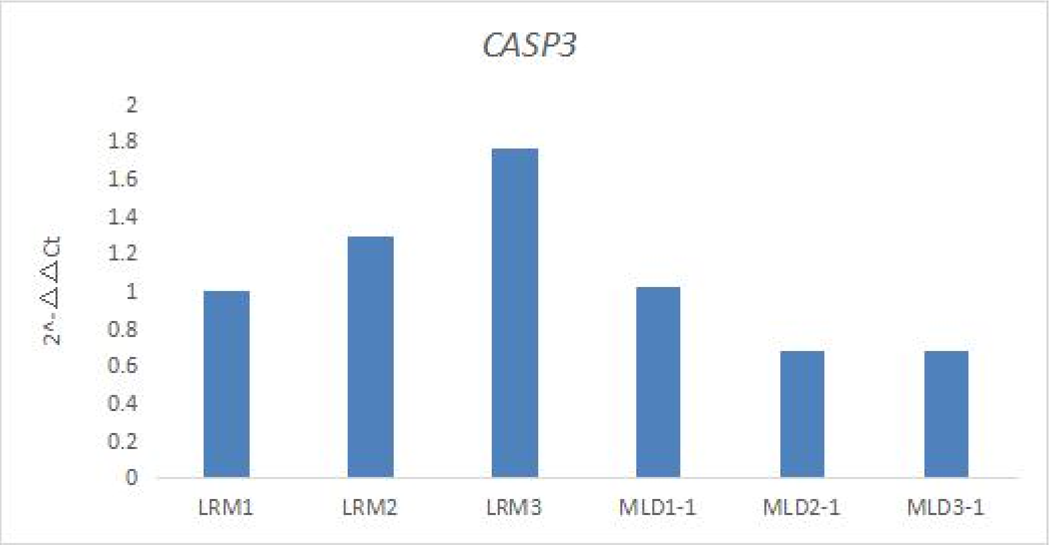

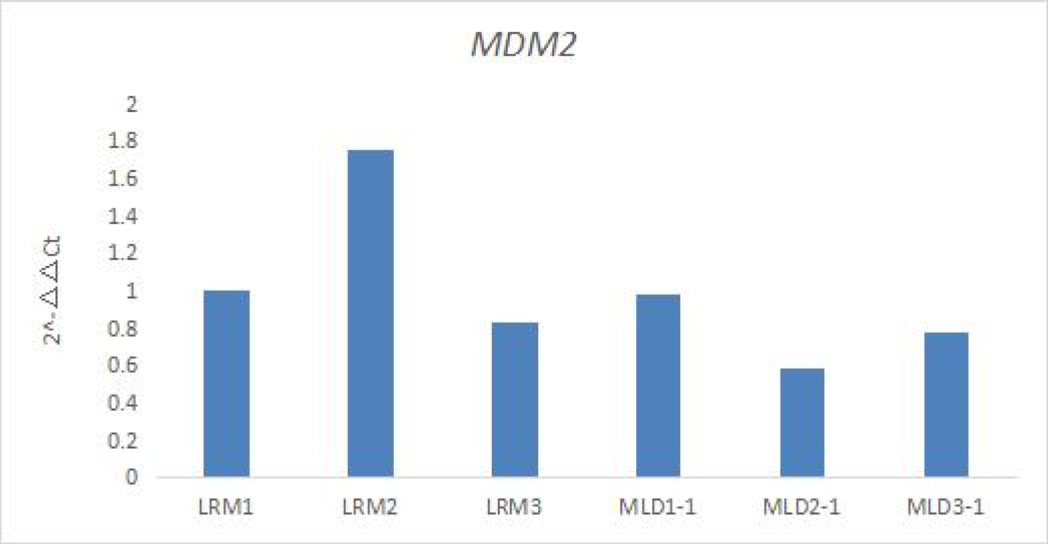

MDM2
2^-△△Ct
2
1.8
1.6
1.4
1.2
1
0.8
0.6
0.4
0.2
0
LRM1
LRM2
LRM3
MLD1-1
MLD2-1
MLD3-1

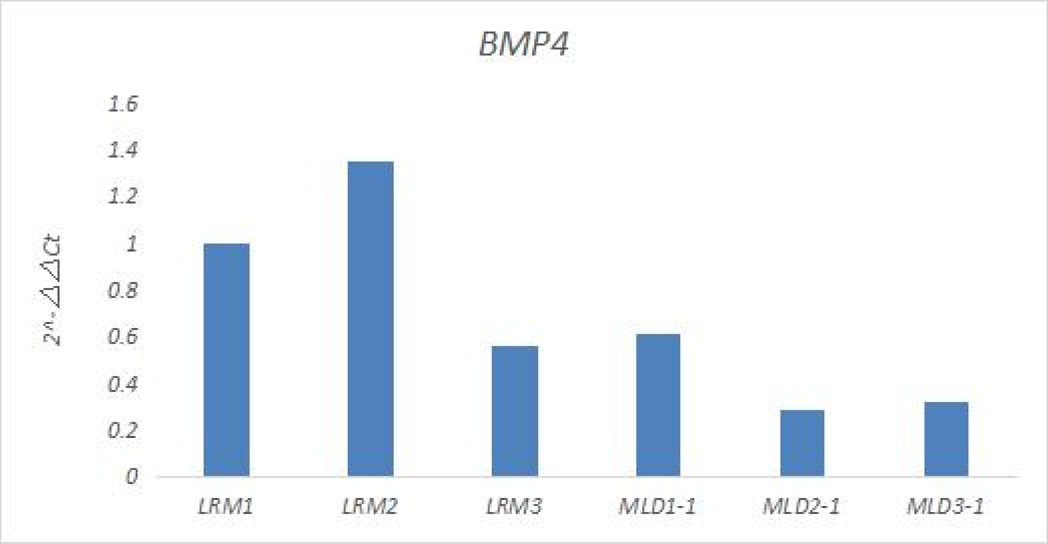

BMP4
2^-△△Ct
1.6
1.4
1.2
1
0.8
0.6
0.4
0.2
0
LRM1
LRM2
LRM3
MLD1-1
MLD2-1
MLD3-1

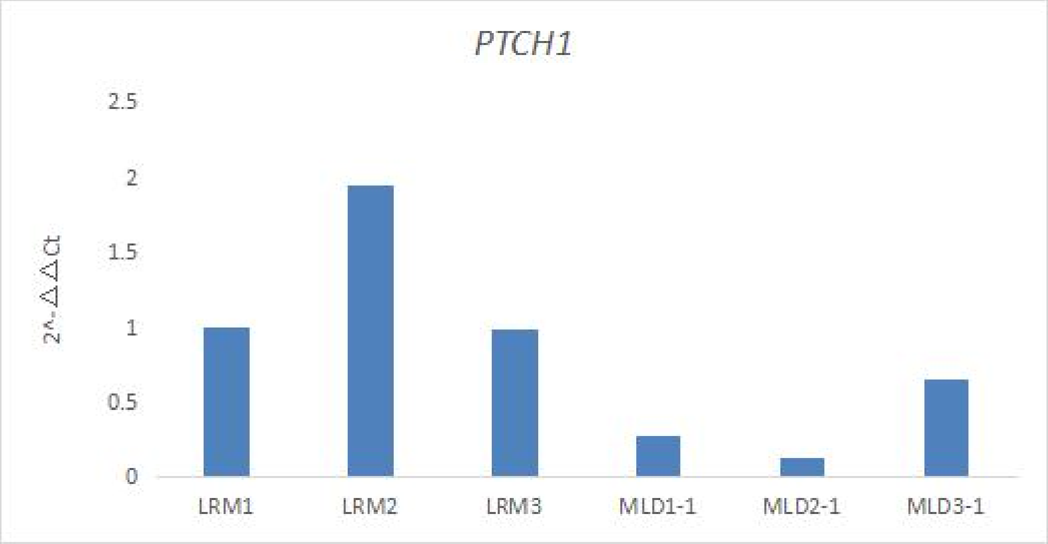

PTCH1
2^-△△Ct
2.5
2
1.5
1
0.5
0
LRM1
LRM2
LRM3
MLD1-1
MLD2-1
MLD3-1
